# Pre-Symptomatic Detection of Influenza with Taste

**DOI:** 10.1021/acscentsci.5c01933

**Published:** 2025-10-27

**Authors:** Shichao Lin, Chaoyong Yang

**Affiliations:** † MOE Key Laboratory of Spectrochemical Analysis & Instrumentation, State Key Laboratory of Physical Chemistry of Solid Surfaces, Innovation Laboratory for Sciences and Technologies of Energy Materials of Fujian Province (IKKEM), Key Laboratory for Chemical Biology of Fujian Province, Department of Chemical Biology, College of Chemistry and Chemical Engineering, 12466 Xiamen University, Xiamen 361005, China; ‡ Institute of Molecular Medicine, School of Medicine, Shanghai Jiao Tong University, Shanghai 200120, China

## Abstract

A viral neuraminidase-specific sensor has been developed to directly detect influenza via the tongue.

## Graphical abstract

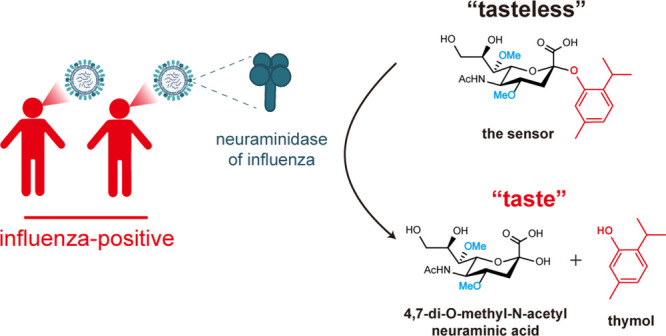

Influenza, a contagious respiratory disease caused by influenza viruses, is responsible for more than 300,000 deaths worldwide each year.^1^ In history, influenza has caused some of the deadliest pandemics, such as the “Spanish flu”,^3^ the swine flu pandemic,^4^ and the H5N1 zoonotic outbreak.^5^ An important lesson from the influenza pandemics and the recent COVID-19 pandemic is the need for early quarantine and isolation of patients to prevent spreading of viruses. However, quarantine is challenging with influenza due to presymptomatic influenza-virus transmission. Therefore, presymptomatic detection of influenza in massive screening is critical for successful control of future influenza outbreaks, which requires diagnostics with high accuracy, high specificity, high speed, and low cost.

In this issue of *ACS Central Science*, Meinel and co-workers meet this challenge by developing a viral neuraminidase-specific sensor that detects influenza by taste.

The authors take advantage of the virus’s need for neuraminidase to cleave α-glycosidic bonds of sialic acid receptors on the host cell membrane and design an *N*-acetylneuraminic acid-thymol derivative (the sensor) to detect neuraminidase, a surface protein of predominant circulating types A and B of influenza.^6^ The sensor specifically responds to neuraminidase at clinically relevant concentrations and rapidly releases thymol within 10 min (Figure 1A). The released thymol is a model taste molecule that can be directly detected by the tongue. The new sensor provides an ideal solution to presymptomatic influenza detection, enabling rapid, cost-effective, and massive screening with high sensitivity and high specificity. To improve the selectivity of the sensor toward viral neuraminidase rather than bacterial neuraminidase, Meinel et al. modified two methyl groups on the sugar moiety (O4 and O7) of the *N*-acetylneuraminic acid-thymol, as further validated by structural analysis and molecular docking. In addition, the sensor shows good stability at various conditions (e.g., −20, 4, and 25 °C at 60% relative humidity for 4 weeks), which is beneficial for storage and transport during future epidemics and pandemics.

**Figure 1:**
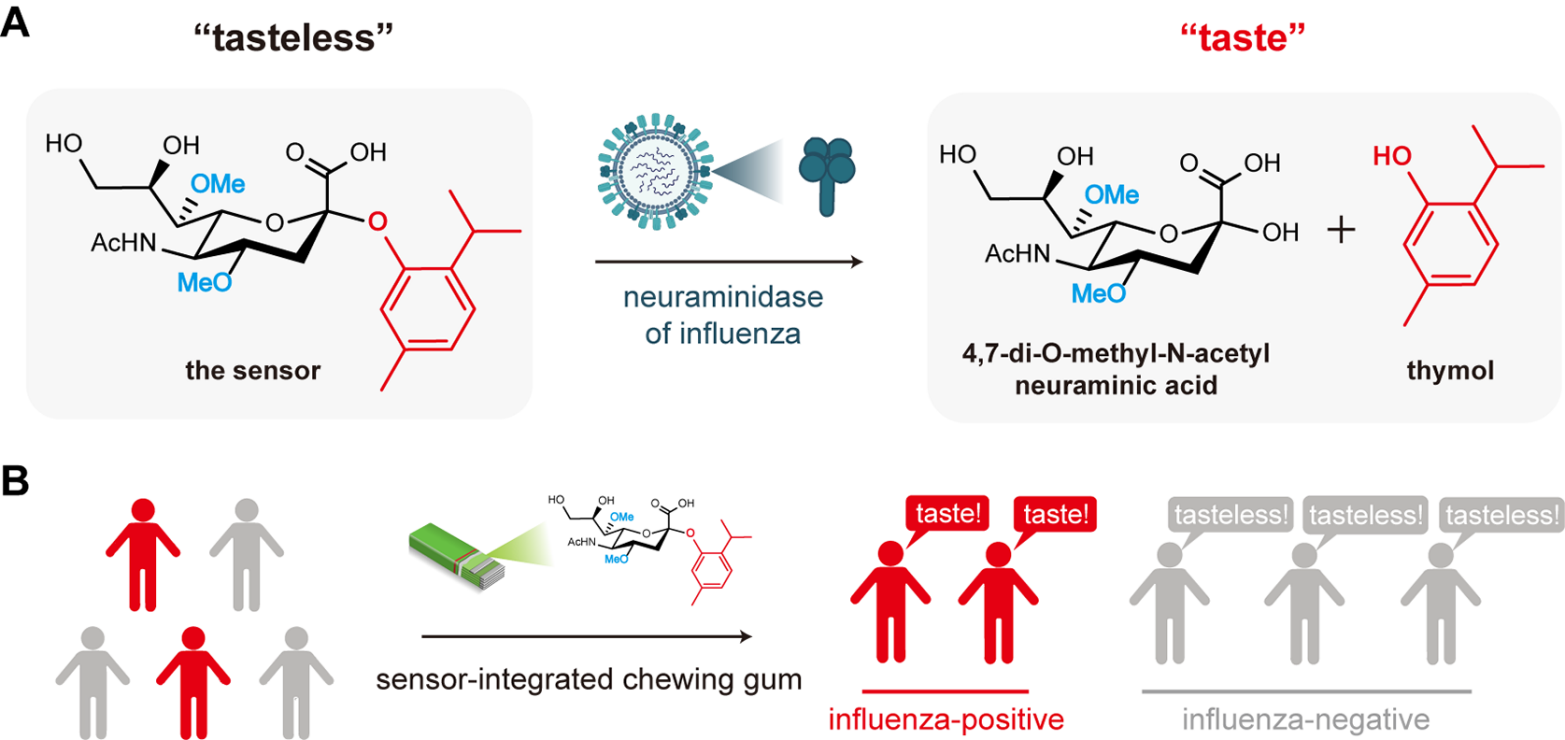
(A) Schematic illustration of the taste-based presymptomatic detection of influenza. (B) A scenario of the application of the taste-based sensor in early quarantine of influenza patients.

The history of influenza detection is a fascinating story that evolves from diagnosis based on symptoms alone to rapid identification of specific viral strains at the genetic level.^7,8^ Methods such as direct observation of the virus or serological test of antibodies based on the immune response were time-consuming and delayed the timely isolation and treatment of patients. To enable rapid influenza diagnostic tests, methods detecting influenza virus-associated antigens in throat swabs or nasal swabs were developed, which have now been translated into point-of-care testing (PoCT) that can be performed by anyone, anywhere, and at any time. However, these antigen-based tests suffer from low to moderate sensitivity, leading to false negatives. In recent decades, polymerase chain reaction (PCR) has become the gold standard of virus detection. The selective amplification of virus RNA allows accurate detection of influenza with high sensitivity and high specificity, and even accurate discrimination between different subtypes of the virus. Nevertheless, PCR-based methods require several hours of detection time and overly expensive equipment and reagents, which make their widespread use challenging, especially in low-income countries.

With the above background of influenza detection, we can now appreciate the contribution of Meinel and co-workers to this field. Their work led to our first reaction—wow, such a brilliant and elegant design!

The developed sensor ranks as a well-rounded player in influenza detection with high sensitivity, high specificity, high speed, and low cost.

However, looking forward, there are still a few concerns that need to be addressed. First, the ongoing mutations of the influenza virus during outbreaks can lead to variations in neuraminidase. It is unclear whether the developed sensor will still be effective against new variants. Second, the commercial application of the sensor may integrate the sensor into chewing gum or thin films to detect the influenza virus in the saliva (Figure 1B). The biosafety of the released *N*-acetylneuraminic acid-thymol derivative needs to be carefully evaluated by animal experiments and even clinical trials before the sensor can be applied. On the positive side, extension of the strategy of taste-based detection of the influenza virus to the presymptomatic detection of other viral or bacterial infections will be highly valuable. We believe that this future direction will have huge impacts on infectious disease control and public health.
